# RXRα Positively Regulates Expression of the Chicken *PLIN1* Gene in a PPARγ-Independent Manner and Promotes Adipogenesis

**DOI:** 10.3389/fcell.2020.00349

**Published:** 2020-05-14

**Authors:** Yuhang Sun, Guiying Zhai, Rui Li, Weinan Zhou, Yumao Li, Zhiping Cao, Ning Wang, Hui Li, Yuxiang Wang

**Affiliations:** ^1^Key Laboratory of Chicken Genetics and Breeding, Ministry of Agriculture and Rural Affairs, Harbin, China; ^2^Key Laboratory of Animal Genetics, Breeding and Reproduction, Education Department of Heilongjiang Province, Harbin, China; ^3^College of Animal Science and Technology, Northeast Agricultural University, Harbin, China

**Keywords:** retinoid X receptor α, PLIN1, chicken, transcriptional regulation, adipogenesis

## Abstract

Perilipin1 (PLIN1), the most abundant lipid droplet (LD)-associated protein, plays a vital role in regulating lipid storage and breakdown in adipocytes. Recently, we found that the overexpression of PLIN1 promotes chicken preadipocyte lipid accumulation. However, the mechanisms by which transcription of the chicken *PLIN1* gene is regulated remain unknown. In this study, we investigated the role of retinoid X receptor α (RXRα) in transcription of the chicken *PLIN1* gene. Notably, reporter gene and expression assays showed that RXRα activates transcription of the chicken *PLIN1* gene in a PPARγ-independent manner. Furthermore, promoter deletion and electrophoretic mobility shift assay (EMSA) analysis revealed that the chicken *PLIN1* gene promoter region (-774/-785) contains an RXRα-binding site. Further study demonstrated that RXRα overexpression promotes differentiation of an immortalized chicken preadipocyte cell line (ICP1), causing a concomitant increase in *PLIN1* transcripts. Taken together, our results show for the first time that RXRα activates transcription of the chicken *PLIN1* gene in a PPARγ-independent manner, which might be at least in part responsible for RXRα-induced adipogenesis.

## Introduction

Obesity is a major risk factor for the development of various diseases such as type 2 diabetes, cardiovascular disease and cancer (Ferguson et al., 2013). Obesity is associated with excess caloric intake and metabolic dysfunctions in adipocytes, leading to excess fat accumulation, which negatively impacts feed conversion efficiency, carcass quality and reproductive performance in broilers (Zhang et al., 2018). Excess calories are stored as fat in lipid droplets (LDs). LDs, intracellular organelles synthesized from the endoplasmic reticulum (ER), are composed of a core of neutral lipids surrounded by a phospholipid monolayer with different associated proteins (Martin and Parton, 2006; Fujimoto and Parton, 2011; Walther and Farese, 2012). LDs are associated with numerous cellular metabolic processes such as energy production; membrane biogenesis; protein modification; and the synthesis of lipoproteins, steroids and other lipid mediators (Fujimoto and Parton, 2011). The storage and hydrolysis of fat are controlled by LD-binding proteins. Among LD-associated proteins, perilipin (PLIN) family proteins are the best characterized and play important roles in regulating lipid metabolism (Greenberg et al., 1991; Brasaemle, 2007; Ducharme and Bickel, 2008; Kimmel et al., 2010; Greenberg et al., 2011).

Perilipin1 (PLIN1) is the most abundant LD-associated protein in adipocytes and plays dual roles in controlling both basal and β-adrenergic receptor agonist-stimulated lipolysis in adipocytes (Brasaemle et al., 2009). Consistent with findings in mammals, PLIN1 also plays a crucial role in maintaining lipid homeostasis in chickens. Our previous data showed that PLIN1 expression is higher in the adipose tissue of fat broilers than in that of lean broilers at 7 weeks of age (Wang et al., 2011) and that LDs in chicken adipocytes are surrounded by PLIN1 at different time points postdifferentiation (Qin et al., 2016). Furthermore, under basal conditions, the overexpression of PLIN1 promotes chicken preadipocyte lipid accumulation (Miyoshi et al., 2006; Miyoshi et al., 2007; Miyoshi et al., 2008; Zhou et al., 2012).

In mammals, the *PLIN1* gene is transcriptionally regulated by numerous factors including peroxisome proliferator-activated receptor γ (PPARγ) (Arimura et al., 2004), estrogen receptor-related receptor α (ERRα) (Akter et al., 2008), liver X receptor (LXR) (Stenson et al., 2011), constitutive coactivator of PPARγ (CCPG) (Li et al., 2007), tribbles homolog 3 (TRB3) (Takahashi et al., 2008), tumor necrosis factor-α (TNF-α) (Souza et al., 2003), RAR-related orphan receptor α (RORα) (Ohoka et al., 2009), docosahexaenoic acid (DHA) (Lecchi et al., 2013), 17 β-estradiol (Wohlers and Spangenburg, 2010), acylation stimulating protein (ASP) (Wu et al., 2011), serum amyloid A (SAA) (Liu et al., 2011), eicosapentaenoic acid (EPA) (Wang et al., 2010) and estrogen receptor α (ERα) (Wend et al., 2013). However, the regulatory mechanisms of chicken *PLIN1* gene transcription remain elusive. In the present study, we uncovered that RXRα positively regulates expression of the chicken *PLIN1* gene in a PPARγ-independent manner and promotes adipogenesis.

## Materials and Methods

### Ethics Statement

All animal work was conducted in accordance with the guidelines for the care and use of experimental animals established by the Ministry of Science and Technology of the China (approval no. 2006-398) and approved by the Institutional Biosafety Committee of Northeast Agricultural University (Harbin, China). Plasmid construction and transfection were performed according to the directions of the Regulation on Safety Administration of Agricultural Genetically Modified Organisms (RSAGMO) established by the China (revised version 2017).

### Cell Culture and Differentiation

Abdominal adipose tissue was excised from 12-day-old Arbor Acres birds and digested. Primary chicken preadipocytes and an immortalized chicken preadipocyte cell line (ICP1) were cultured and differentiated according to the methods of our laboratory (Wang et al., 2008; Shang et al., 2014; Wang et al., 2017). Briefly, adipose tissue was washed by pre-warmed PBS supplemented with penicillin (100 units/ml) and streptomycin (100 μg/mL), cut with surgical scissors, and digested in 2 mg/mL collagenase type I (Invitrogen, Grand Island, NY, United States) with shaking for 65 min at 37°C. After digestion, the cell suspension was filtered through a 20-μm mesh and centrifuged at 300 *g* for 10 min at room temperature (22°C) to separate the stromal-vascular fractions from undigested tissue debris and mature adipocytes. Stromal-vascular cells (including preadipocytes) or ICP1 cells were seeded at a density of 1 × 10^6^ cells/cm^2^ in Dulbecco’s modified Eagle’s medium/F12 medium (Invitrogen) with 5% fetal bovine serum (FBS, Invitrogen) and maintained at 37°C in a humidified atmosphere of 5% CO_2_ until confluency (day 4). The cells were then trypsinized (0.25% trypsin + 0.04% EDTA) and passaged. DF-1 chicken fibroblast cells (Harbin Veterinary Research Institute, Heilongjiang, China) were maintained in Dulbecco’s modified Eagle’s medium (DMEM, Invitrogen) supplemented with 5% FBS at 37°C in a humidified atmosphere of 5% CO_2_.

One day after propagation (day 5), when the cells had reached 50% confluence, primary chicken preadipocytes and ICP1 cells were induced by growth in complete medium containing 160 μM sodium oleate (Sigma-Aldrich, St. Louis, MO, United States) for differentiation. Subsequently, the medium was removed every 24 h and replaced with fresh medium containing DMEM/F12 supplemented with 10% FBS and 160 μM sodium oleate. Preadipocytes and ICP1 cells were differentiated for a total of 72 h.

### RNA Isolation and Quantitative Real-Time RT-PCR

Total RNA was extracted from chicken abdominal fat tissue and cells with TRIzol^®^ Reagent (Invitrogen) following the supplier’s protocol. Total RNA was treated with DNase I (TaKaRa, Dalian, China), and RNA quality was assessed by visualization of the 18S and 28S ribosomal RNA bands on a denaturing formaldehyde agarose gel. Only RNA with a 28S:18S ratio between 1.8 and 2.1 was used for reverse transcription. Reverse transcription was performed according to the directions of the ImProm-II^TM^ Reverse Transcription System (Promega, Madison, WI, United States).

Quantitative real-time RT-PCR was used to analyze gene expression levels. Expression levels of β*-actin* and the TATA-box binding protein (*TBP*) gene were used as internal references. Quantitative RT-PCR was performed using FastStart Universal SYBR Green Master (ROX) (Roche Life Science, Indianapolis, IN, United States) on a 7500 Real-Time PCR System (Applied Biosystems, Foster City, CA, United States). From each 10-μL reaction, 1 μL of product was amplified. The following PCR conditions were used: incubation for 1 cycle at 95°C for 10 min, followed by 40 cycles of 95°C for 15 s and 60°C for 1 min. Dissociation curves for each PCR were analyzed using Dissociation Curve 1.0 software (Applied Biosystems) to detect and eliminate possible primer-dimer artifacts. The relative level of target gene expression, as determined with ABI software, was calculated using the comparative 2^–Δ^ ^Δ^ ^*Ct*^ method for relative quantification. The sequences of the primers used to analyze gene expression levels are shown in Table 1.

**TABLE 1:** PCR primers used in this study.

Primer name	Sequence (5’-3’)	Length
Cloning PLIN-1992/-11	F: cgg ggtacc TGGGCTGTCTCAGCAAGTACAGTCT	1982 bp
Cloning PLIN-1834/-11	F: gg ggtacc GCTGGGGGCTAGCAGTTAAATGTACC	1824 bp
Cloning PLIN-1307/-11	F: cgg ggtacc GCAGAATGGTAAGTGAGATAAGTAATCT	1297 bp
Cloning PLIN-838/-11	F: g ggtacc CTGGTGTCATGCCTGTTCACCGTGG	828 bp
Cloning PLIN-689/-11	F: cgg ggtacc GTTAATGCAGGGCTGTGGACAAG	679 bp
Cloning PLIN-470/-11	F: cgg ggtacc TGCTGGTCCAAGTGAGTAAG	460 bp
Cloning PLIN-246/-11	F: g ggtacc TCCTCCTCTTCTCCCTAGCCTTGGT	236 bp
Cloning PLIN-123/-11	F: g ggtacc TCCCACAAGATGAGAACCTG	113 bp
	R: c ctcgag GTGTGGTGTTGGGGCACTACTACACC	
Cloning PLIN mut-838/-11	F: GTGAGCAGGCTGCTAAGCTTTGTCCCACTGTCT	828 bp
	R: AGACAGTGGGACAAAGCTTAGCAGCCTGCTCAC	
Cloning RXRα CDS	F: cg gaattc TGGACACCAAACACTTCCTGCCACT	1617 bp
	R: c ctcgag TTAGATGCAGCAGTGACAGCGAACG	
qRT-PCR *PLIN1*	F: GCCAAGGAGAACGTGCT	142 bp
	R: TCACTCCCTGCTCATAGACC	
qRT-PCR *RXR*α	F: GATGCGAGACATGCAGATG	163 bp
	R: GTCGGGGTATTTGTGCTTG	
qRT-PCR *PPAR*γ	F: GTGCAATCAAAATGGAGCC	170 bp
	R: CTTACAACCTTCACATGCAT	
qRT-PCR *AP2*	F: ATGTGCGACCAGTTTGT	143 bp
	R: TCACCATTGATGCTGATAG	
qRT-PCR *TBP*	F: GCGTTTTGCTGCTGTTATTATGAG	122 bp
	R: TCCTTGCTGCCAGTCTGGAC	

### Plasmid Construction

The chicken *PLIN1* promoter and its subsequent 5’ truncation construct were generated by PCR from chicken genomic DNA using different forward primers and the same reverse primer as shown in Table 1 and then subcloned into the pGL3-Basic vector (Promega). Site-directed mutagenesis was performed with a QuickMutation Site-Directed Mutagenesis Kit (Beyotime Institute of Biotechnology, Jiangsu, China). The site-mutated promoter was cloned into the pGL3-Basic vector. A chicken RXRα expression plasmid containing the coding region of the chicken *RXR*α gene (GenBank Accession No. XP_003642339.1) was constructed by RT-PCR from chicken abdominal fat tissue total RNA and cloned into the pCMV-Myc vector (Clontech, Mountain View, CA, United States). All primers used are shown in Table 1, and all final constructs were confirmed by DNA sequencing. The pCMV-HA-PPARγ plasmid was constructed and preserved by our laboratory.

### Western Blot Analysis and Electrophoretic Mobility Shift Assay

To prepare nuclear extracts, the pCMV-Myc-RXRα expression vector was transfected into DF-1 cells. After 48 h of transfection, nuclear extracts were collected using NE-PER extraction reagents (Pierce, Waltham, MA, United States). Part of the nuclear extracts was used for Western blotting. After being mixed with 6× denaturing loading buffer and boiled for 5 min, nuclear proteins were separated by 12% sodium dodecyl sulfate-polyacrylamide gel electrophoresis and transferred to an Immun-Blot polyvinylidene fluoride membrane (Millipore, Billerica, MA, United States). Western blotting was performed using anti-Myc bodies with ECL (Beyotime Institute of Biotechnology).

Nuclear extracts were incubated with a biotin-labeled *PLIN1* promoter DNA probe (5′-3′ Biotin) for 20 min at room temperature and then separated by electrophoresis on a 5% non-denaturing polyacrylamide gel with 0.5 × TBE running buffer. DNA-protein complexes were transferred onto nylon membranes (Pierce) and then crosslinked for 1 min with a UV crosslinker. The signal was detected with a Chemiluminescent EMSA Kit (Beyotime Institute of Biotechnology) according to the manufacturer’s instructions. For the competition assay, nuclear extracts were incubated with unlabeled probes (Invitrogen) for 10 min at room temperature before the addition of biotin-labeled oligonucleotide. For the supershift assay, protein-DNA complexes were incubated with 1 μg of antibody specific to the Myc tag (Abcam, Cambridge, MA, United States) for 30 min at room temperature before electrophoresis. Sequences of the probes used for EMSA are shown in Table 2.

**TABLE 2:** Sequences of probes used for EMSA.

Gene name	Probe name	Sequence (5′-3′)
*PLIN1*	-59/-11	GCCCAGCCCAGAGGTGGGGCCTAGGTGTAGTAGTGCCCCAACACCACAC
	-97/-46	GCTGTTTGCCCGGTTTCCCCAGCAACTCATGCCCCCCAGCCCAGCCCAGAGG
	-153/-85	GACGTATGGGGATGATTTTGCAGCCATCCATCCCACAAGATGAGAACCTGTG GGGAGCTGTTTGCCCGG
	-791/-766	AGGCTGCTGCCCTTTGTCCCACTGTC
	Mut-791/-766	AGGCTGCTAAGCTTTGTCCCACTGTC

*Unlabeled probe sequence is identical to labeled probe sequence. N (non) is added before the labeled probe, for example, N-59/-11, N-97/-46, N-153/-85, and N-791/-766.*

### Luciferase Reporter Gene Assay

DF-1 cells at 70–80% confluence were washed with PBS and transiently transfected using Lipofectamine 2000 reagent (Invitrogen). Forty-eight hours later, luciferase activity was measured using a Dual-Luciferase Reporter Assay System (Promega) according to the manufacturer’s instructions on a FB12 luminometer (Berthold Detection Systems, Pforzheim, Germany). Firefly luciferase (Fluc) activity was normalized to Renilla luciferase (Rluc) activity.

### Oil Red O Staining and Extraction Assay

The differentiated ICP1 cells were washed with PBS and fixed with 4% paraformaldehyde for 30 min at 4°C. After being washed with PBS and distilled water twice, the cells were stained with oil red O working solution (oil red O dye in 60% isopropanol) at room temperature for 15 min. Cells were then washed immediately with ddH_2_O and analyzed under a microscope (Leica, Wetzlar, Germany).

To quantitatively measure lipid accumulation, an oil red O extraction assay was performed. Briefly, after removing the staining solution, oil red O was extracted by the addition of 1 mL of 100% (v/v) isopropyl alcohol, and the absorbance at 510 nm was measured with a spectrophotometer (Ultrospec 1000, Pharmacia Biotech, Canton, MA, United States).

### Statistical Analysis

All data are presented as the mean ± SEM. Differences between groups were analyzed using unpaired Student’s *t-tests* conducted with GraphPad Prism 5. Statistical significance was indicated when ^∗^*P* < 0.05, ^∗∗^*P* < 0.01.

## Results

### Expression of the Chicken *PLIN1* and *RXR*α Genes During Preadipocyte Differentiation

**FIGURE 1:**
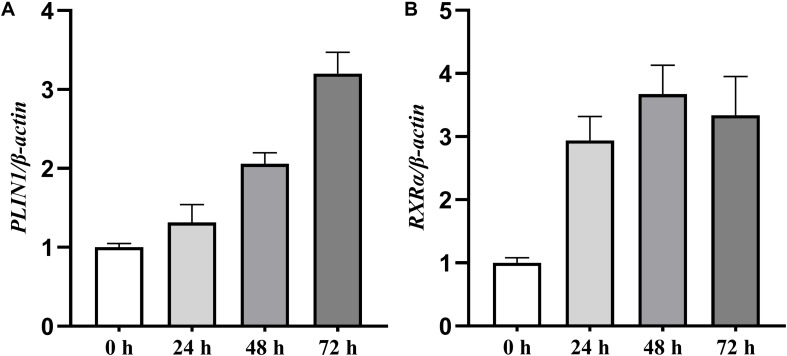
Expression levels of the *PLIN1* and *RXR*α genes during primary chicken preadipocyte differentiation. The differentiation of primary chicken preadipocytes at 50% confluence was induced by the addition of sodium oleate in fresh medium that was changed every 24 h for 72 h of culture. The cells were harvested after 0, 24, 48, and 72 h of differentiation, and real-time RT-PCR was performed. The chicken β*-actin* gene was used as an internal control. The data are the means of three individual values ±SEM (*n* > 3 independent experiments). **P < 0.01. **(A)** The expression profiles of the chicken *PLIN1* genes during the primary chicken preadipocyte differentiation. **(B)** The expression profiles of chicken *RXR*α gene during the primary chicken preadipocyte differentiation.

We first characterized the expression profiles of the chicken *PLIN1* and *RXR*α genes during the adipogenesis of primary chicken preadipocytes. During preadipocyte differentiation, the mRNA expression levels of chicken *PLIN1* gene were gradually elevated; the expression level of chicken *RXR*α gene increased rapidly after preadipocyte induction and maintained high expression throughout the differentiation process (Figures 1A,B). These results suggest that PLIN1 and RXRα play a role in chicken preadipocyte adipogenesis.

### PPARγ-Independent Transcriptional Activation of the Chicken *PLIN1* Gene by RXRα

**FIGURE 2:**
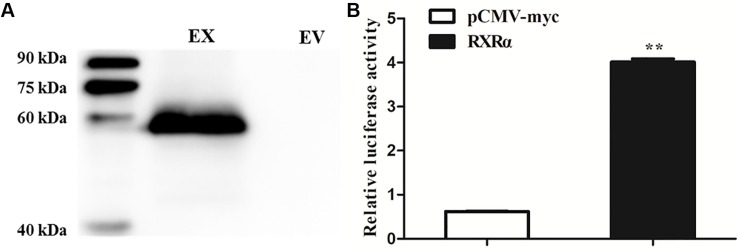
Transcriptional activation of the chicken *PLIN1* gene by RXRα. **(A)** Western blotting was performed to analyze chicken RXRα expression. DF-1 cells were transfected with the pCMV-Myc-RXRα or pCMV-Myc vector. Nuclear extracts were harvested 48 h after transfection and immunoblotted with a Myc-specific antibody; EX: pCMV-Myc-RXRα, EV: pCMV-Myc empty vector. **(B)** Activity of the chicken *PLIN1* promoter. DF-1 cells were cotransfected with the chicken *PLIN1* reporter plasmid (pGL3-Plin-1992/-11) and pCMV-Myc-RXRα (white bar) or pCMV-Myc vector (black bar). After 48 h of transfection, promoter activity was assayed and is expressed as relative luciferase activity (Fluc/Rluc). All data are expressed as the mean ± SEM (*n* > 3 independent experiments). **P < 0.01.

To determine whether RXRα regulates transcription of the chicken *PLIN1* gene, luciferase assays with reporter genes were performed. First, Western blotting was developed to confirm that the RXRα protein was overexpressed after DF-1 cells were transfected with the pCMV-Myc-RXRα plasmid (Figure 2A). Then, DF-1 cells were transiently cotransfected with the *PLIN1* gene promoter reporter plasmid (pGL3-PLIN1-1992/-11) and pCMV-Myc-RXRα, and luciferase activity was measured. DF-1 cells transfected with the pCMV-Myc vector were used in the control group. As shown in Figure 2B, chicken *PLIN1* promoter activity was higher in the RXRα overexpression group than in the control (*P* < 0.01).

**FIGURE 3:**
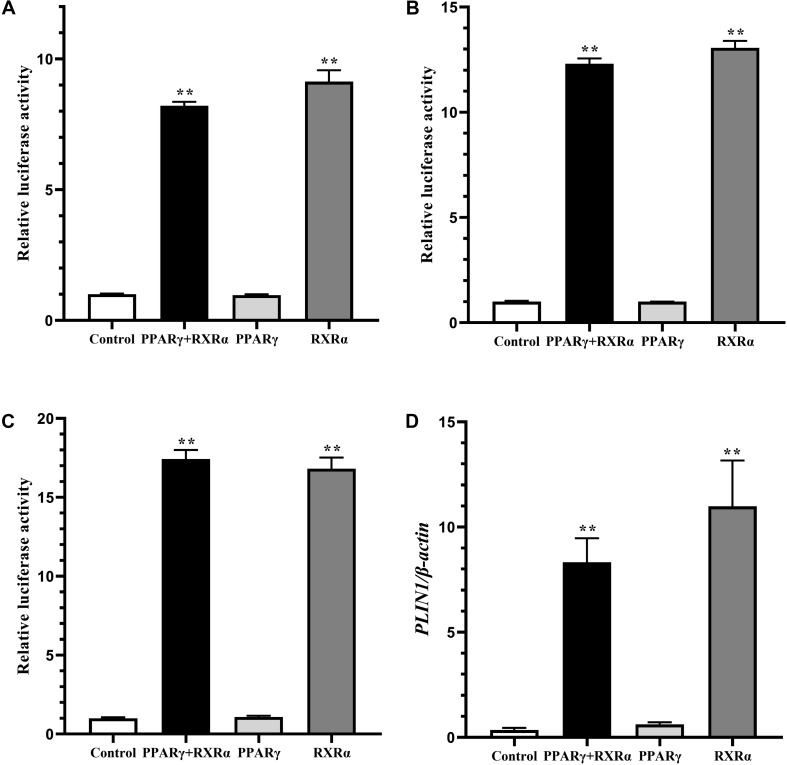
Effect of PPARγ/RXRα overexpression on the promoter activity and expression of the chicken *PLIN1* gene. **(A)** Luciferase activity assay in chicken DF-1 cells. DF-1 cells were cotransfected with the chicken *PLIN1* reporter plasmid (pGL3-Plin-1992/-11) and pCMV-Myc-RXRα/pCMV-PPARγ (PPARγ + RXRα), pCMV-PPARγ alone (PPARγ), pCMV-Myc-RXRα alone (RXRα), or pCMV-Myc plasmid (Control). After 48 h of transfection, luciferase reporter activity was assayed and is expressed as the relative luciferase activity (Fluc/Rluc). **(B)** Luciferase activity assay in chicken DF-1 cells after troglitazone treatment. DF-1 cell were cotransfected with the above plasmids and the PPARγ agonist troglitazone at 5 μM was added at the same time. After 48 h of transfection, luciferase reporter activity was assayed and expressed as relative luciferase activity (Fluc/Rluc). **(C,D)** Luciferase activity and *PLIN1* gene expression assay in chicken preadipocytes. The chicken preadipocytes were induced by replacing the induction medium containing oleic acid at 80–90% confluence. After 24 h of induction, cotransfection of the above plasmids was performed. Forty-eight hours later, luciferase reporter activity was assayed **(C)**, and the mRNA levels of chicken *PLIN1* were determined by real-time RT-PCR and normalized to chicken β*-actin* mRNA levels **(D)**. All data are expressed as the mean ± SEM. *n* ≥ 3, **P < 0.01.

In mammals, RXRα upregulates expression of the *PLIN1* gene in the form of only a PPARγ2/RXRα heterodimer (Arimura et al., 2004). To determine whether a similar mechanism occurs in chickens, DF-1 cells and postdifferentiated adipocytes were cotransfected with pGL3-PLIN1-1992/-11 and pCMV-Myc-RXRα/pCMV-PPARγ, pCMV-PPARγ alone, or pCMV-Myc-RXRα alone. Cotransfection with PPARγ/RXRα heterodimers and RXRα alone increased the promoter activity of the *PLIN1* gene in DF-1 cells, regardless of the presence or absence of troglitazone, a PPARγ ligand (*P* < 0.01) (Figures 3A,B). Similarly, in adipocytes differentiated for 24 h, transfection with the PPARγ/RXRα heterodimer and RXRα alone significantly increased the promoter activity and intracellular mRNA expression of the *PLIN1* gene (*P* < 0.01) (Figures 3C,D). However, transfection with PPARγ alone had no effect on the promoter activity and mRNA expression of the chicken *PLIN1* gene (Figures 3A–D). These results indicate that chicken *PLIN1* expression can be activated by RXRα without PPARγ.

To further verify the distinctive mechanism by which the chicken *PLIN1* gene is regulated by RXRα, we investigated the effect of PPARγ knockdown on RXRα-induced transcriptional activation of the chicken *PLIN1* gene using RNAi. The sh-PPARγ and control (nc-PPARγ) vectors, whose construction and confirmation were reported in our previous study (Wang et al., 2011), were cotransfected into DF-1 cells with pCMV-Myc-RXRα and pGL3-PLIN1-1992/-11. Then, mRNA expression of the chicken *PPAR*γ and *PLIN1* genes and luciferase activities were measured. As shown in Supplementary Figure S1, endogenous chicken *PPAR*γ gene expression decreased after sh-PPARγ transfection (*P* < 0.05, Supplementary Figure S1A), but there was no difference in the expression (Supplementary Figure S1B) and promoter activity (Supplementary Figure S1C) of the chicken *PLIN1* gene in the presence and absence of *PPAR*γ. These results indicate that RXRα could activate expression of the chicken *PLIN1* gene in a PPARγ-independent manner.

### Determination of the Region of the Chicken *PLIN1* Gene Promoter Involved in RXRα-Induced Transcriptional Activation

**FIGURE 4:**
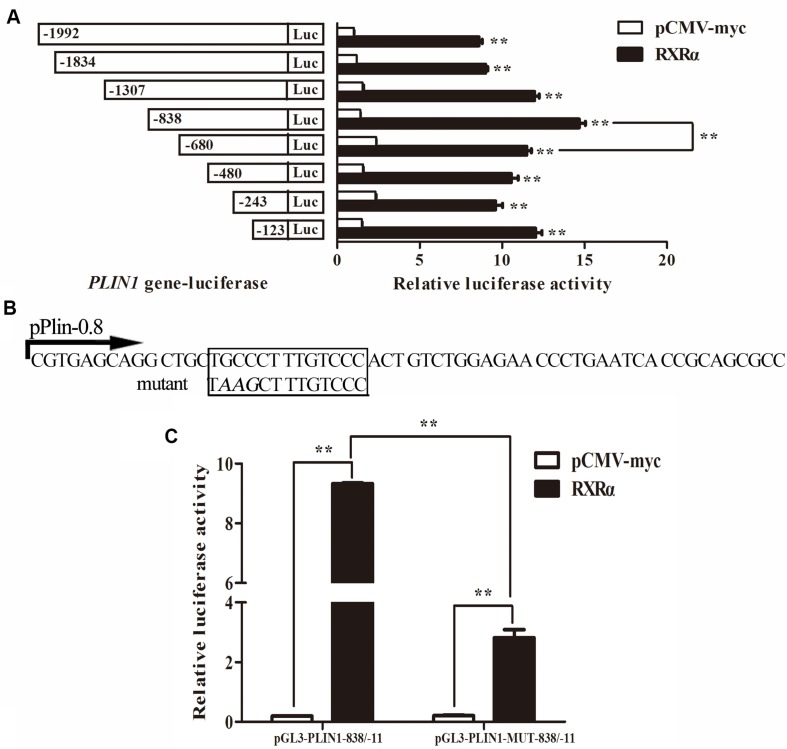
Identification of the chicken *PLIN1* promoter region involved in RXRα-mediated *PLIN1*regulation. **(A)** Regulatory effects of RXRα on 5′ chicken *PLIN1* promoter truncation mutants. DF-1 cells were cotransfected with reporter plasmids containing 5′ chicken *PLIN1* promoter truncation mutants (pGL3-PLIN1-1992/-11, pGL3-PLIN1-1834/-11, pGL3-PLIN1-1307/-11, pGL3-PLIN1-838/-11, pGL3-PLIN1-680/-11, pGL3-PLIN1-480/-11, pGL3-PLIN1-243/-11 and pGL3-PLIN1-123/-11) and the pCMV-Myc-RXRα or pCMV-Myc vector. After 48 h of transfection, luciferase reporter activity was measured. **(B)** Sequences of the mutant chicken *PLIN1* promoter reporter plasmids. The putative PPARγ:RXRα-binding site is boxed. The mutated sequence in the binding site is indicated by italic letters. **(C)** Effects of mutation of the predicted RXRα-binding sites on RXRα-mediated positive activation of the *PLIN1* promoter. DF-1 cells were cotransfected with wild-type (pGL3-PLIN1-838/-11) or mutant (pGL3-PLIN1-MUT-838/-11) plasmid and the pCMV-Myc-RXRα or pCMV-Myc vector. After 48 h of transfection, promoter activity was assayed and is expressed as relative luciferase activity (Fluc/Rluc). All data are expressed as the mean ± SEM (*n* > 3 independent experiments). **P < 0.01.

To identify the region of the chicken *PLIN1* gene promoter involved in RXRα-induced transcriptional regulation, DF-1 cells were cotransfected with luciferase reporter gene plasmids containing serially truncated chicken *PLIN1* gene promoter sequences and the pCMV-Myc-RXRα or control (pCMV-Myc) vector. As shown in Figure 4A, all the chicken *PLIN1* promoters, even the promoter truncated to -123/-11, could be activated by the expression of RXRα compared with promoter activity in the control (*P* < 0.01). The promoter construct from -838/-11 had the highest promoter activity, while promoter activity decreased significantly as the *PLIN1* promoter was truncated from −838 bp to −680 bp (*P* < 0.01, Figure 4A). These results suggest that the sites positively regulated by RXRα are in the -838/-680 and -123/-11 regions of the chicken *PLIN1* gene promoter.

Furthermore, when we predicted transcription factor-binding sites of the chicken *PLIN1* gene promoter with JASPAR^1^, we found a putative PPARγ:RXRα-binding site (-785/-774) in the -838/-680 region. To define the function of this predicted RXRα-binding site, a pGL3-PLIN1-MUT-838/-11 promoter reporter construct was generated by site-directed mutagenesis using DNA synthesis (Figure 4B). Then, luciferase assays were carried out, and the promoter activities of pGL3-PLIN1-MUT-838/-11 and its corresponding wild-type promoter reporter construct, pGL3-PLIN1-838/-11, in the presence or absence of RXRα were compared. Mutation of three bases (*GCC* to *AAG*) caused a 69.86% decline in chicken *PLIN1* promoter activity with the expression of RXRα compared to that observed with the wild-type reporter (*P* < 0.01) (Figure 4C), suggesting that the RXRα-binding site (-785/-774) is required for RXRα-mediated positive activation of the chicken *PLIN1* promoter.

### The Element on the Chicken *PLIN1* Gene Responsible for RXRα-Mediated Transcriptional Regulation

**FIGURE 5:**
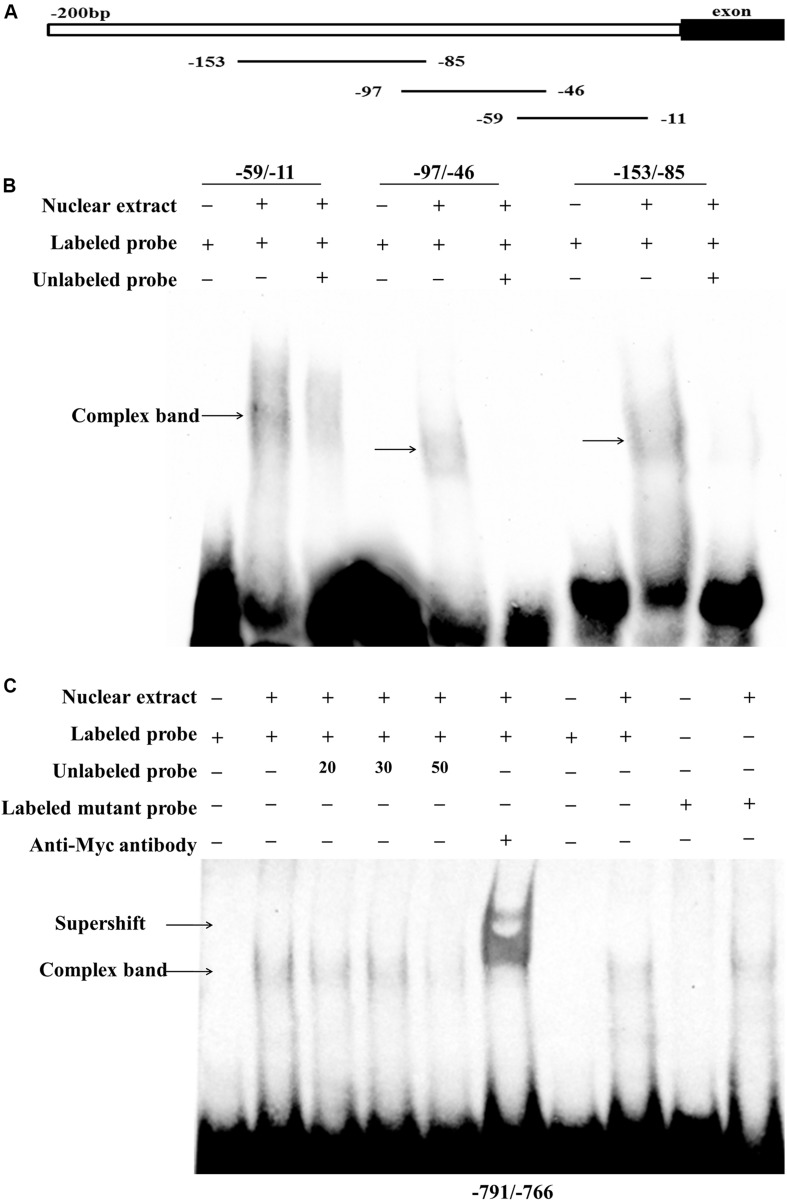
Analysis of RXRα-binding sites in the chicken *PLIN1* promoter using EMSA. **(A)** Schematic diagram of synthesized probes in the -123/-11 region. **(B)** Analysis of the binding affinity of -123/-11 of the *PLIN1* gene promoter to RXRα. A 50-fold molar excess of unlabeled double-stranded DNA fragments derived from the *PLIN1* gene promoter was used in competition assays (lanes 3, 6, 9). **(C)** Analysis of the binding affinity of -785/-774 of the *PLIN1* gene promoter to RXRα. The probe corresponding to -791/-766 was synthesized and labeled with biotin. Nuclear extracts were prepared from DF-1 cells transfected with the pCMV-Myc-RXRα plasmid. Competition EMSA was carried out with a 20-, 30-, and 50-fold molar excess of unlabeled probe (lanes 3-5). A supershift assay was performed with anti-HA antibody (lane 6). The Mut-791/-766 probe, whose binding site was mutated from *GCC* to *AAG*, was incubated with RXRα nuclear extract (lane 10). *n* ≥ 3 independent experiments.

To confirm whether these two putative regions of the chicken *PLIN1* gene promoter, -785/-774 and -123/-11, are directly recognized by RXRα, an electrophoretic mobility shift assay (EMSA) was performed with recombinant nuclear RXRα protein. First, to assess the -123/-11 region, three labeled probes (-59/-11, -97/-46, and -153/-85) were designed. Two adjacent probes overlapped fragments of at least 10 bp (Figure 5A). Three single-shifted DNA-protein complexes were observed in the presence of different labeled fragments and the nuclear RXRα protein (Figure 5B, lanes 2, 5, and 8). Then, we found that those binding bands almost completely disappeared in the presence of an excess amount of an unlabeled fragment (Figure 5B, lanes 3, 6, and 9). These results suggest multiple RXRα protein-binding sites in the -123/-11 region of the chicken *PLIN1* promoter.

To determine whether the predicted -785/-774 site is truly an RXRα-binding site, a probe corresponding to the -791/-766 sequence and a mutated probe containing a *GCC* to *AAG* mutation in the putative binding site, Mut-791/-766, were generated. The EMSA results showed a single-shifted DNA-protein complex that appeared in the presence of the -791/-766 probe and the RXRα nucleoprotein (Figure 5C, lane 2); the signal for the DNA-protein complex gradually disappeared with the addition of a 20-, 30-, and 50-fold molar excess of unlabeled probe (Figure 5C, lanes 3, 4 and 5), suggesting that the DNA binding is sequence-specific. Then, a supershift assay was performed. With the addition of a specific antibody, a retarded band corresponding to the DNA-protein-antibody complex appeared above the single-shifted DNA-protein complex band (Figure 5C, lane 6), which confirms that RXRα binds specifically to the predicted binding site. In addition, the complex signal was still present after incubation of the Mut-791/-766 probe and RXRα nuclear extract, indicating that mutation of these three bases weakened the binding of DNA to protein, but the protein could still bind DNA, which was consistent with the results of the reporter gene experiment (Figure 5C). These results indicate that the RXRα protein can recognize and bind to a binding site of the chicken *PLIN1* promoter at -785/-774.

### Overexpression of RXRα Promoted Chicken Preadipocyte Differentiation

**FIGURE 6:**
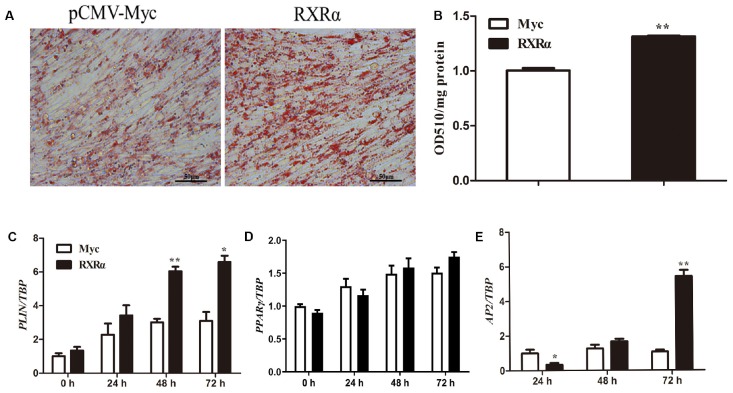
RXRα overexpression promotes chicken preadipocyte differentiation. **(A,B)** The effects of RXRα overexpression on LD accumulation during ICP1 cell differentiation. After 24 h of transfection, cell differentiation was induced by sodium oleate. Oil red O staining **(A)** of ICP1 cells transfected with pCMV-Myc or pCMV-Myc-RXRα was performed after 72 h of differentiation and quantified **(B). (C–E)** The mRNA expression levels of adipocyte differentiation marker genes (*PLIN1*, *PPAR*γ, and *AP2*) during the differentiation of ICP1 cells transfected with either empty pCMV-Myc vector (white bar) or the pCMV-Myc-RXRα vector (black bar). All data are expressed as the mean ± SEM (*n* > 3 independent experiments). *P < 0.05, **P < 0.01.

During primary chicken preadipocyte differentiation, mRNA levels of the chicken *RXR*α gene were elevated (Figure 1B), suggesting that RXRα plays a catalytic role in chicken adipogenesis. To assess this hypothesis, an overexpression experiment was performed by the transfection of pCMV-Myc-RXRα into ICP1 cells. Compared with lipid accumulation in the empty vector-transfected cells, RXRα overexpression significantly increased intracellular lipid accumulation at 72 h postdifferentiation, as indicated by oil red O staining (Figure 6A) and the quantification of the staining intensity (*P* < 0.01, Figure 6B). Consistent with these findings, mRNA expression of the adipogenic marker gene *AP2* increased after 72 h of RXRα overexpression (*P* < 0.01, Figure 6E). Meanwhile, RXRα overexpression increased chicken *PLIN1* mRNA levels after 48 and 72 h of differentiation (*P* < 0.05, Figure 6C), which is consistent with our finding that RXRα positively regulates chicken *PLIN1* promoter activity (Figures 2B, 4A). These results provide evidence that RXRα contributes to chicken preadipocyte differentiation.

## Discussion

Ligand-bound nuclear receptors can regulate target gene expression by binding their response element as a heterodimeric partner with RXRs (Belorusova et al., 2016; Osz et al., 2019) to control a wide range of cellular processes including cell proliferation and lipid metabolism (Lefebvre et al., 2010). Despite their physiological importance, the mode of regulation of *RXR* gene expression has, paradoxically, received little attention (Lefebvre et al., 2010). In this study, we demonstrated that during primary preadipocyte differentiation, mRNA levels of the chicken *RXR*α gene and *PLIN1* gene were all significantly elevated (Figure 1), suggesting that RXRα is involved in chicken adipogenesis and possibly related to the regulation of chicken *PLIN1*.

RXRs regulate gene expression to a considerable extent through their ability to form heterodimers with many other NRs, such as PPARs, LXRs, pregnane X receptor (PXR), farnesoid X receptor (FXR), Nurr1, Nur77, retinoic acid receptors (RARs), vitamin D receptor (VDR), and thyroid receptors (TRs) (Roszer et al., 2013). Studies in mammals suggest that RXRα-mediated transcriptional activation of the *PLIN1* gene is caused by only PPARγ2/RXRα heterodimers rather than RXRα alone or a combination of RXRα and other nuclear receptors (Arimura et al., 2004). Interestingly, in our study, PPARγ alone could not activate the chicken *PLIN1* gene promoter in DF-1 cells in the presence of troglitazone, a PPARγ ligand (Figure 3A). In contrast, PPARγ/RXRα and RXRα alone could activate chicken *PLIN1* promoter activity in both the presence and absence of troglitazone, and there was no obvious difference in the effect between the two groups (Figures 3A–C). Thus, we speculated that RXRα can activate chicken *PLIN1* gene expression via a PPARγ-independent mechanism. Furthermore, with the downregulation of PPARγ expression, the RXRα-induced transcriptional activation and expression of the chicken *PLIN1* gene were not affected (Supplementary Figure S1B), which is consistent with our hypothesis. Evidence suggests that RXRs typically do not function alone but rather serve as partners to other NRs to regulate gene expression (Costa et al., 2010). Therefore, transcriptional activation of the chicken *PLIN1* gene by RXRα may be caused by a combination of RXRα and other nuclear receptors, but not PPARγ. Of course, RXRα may also independently regulate chicken *PLIN1* gene expression in the form of an RXRα homodimer.

Promoter deletion analysis showed that with truncation of the chicken *PLIN1* gene promoter from -1992 to -123 bp, the RXRα-mediated positive regulation of chicken *PLIN1* gene transcription was maintained, suggesting that there are RXRα-binding sites in the -123/-11 region of the *PLIN1* gene promoter (Figure 4A). Meanwhile, as the *PLIN1* promoter was truncated from -838 bp to -680 bp, reporter gene activity decreased significantly, and mutation of the predicted binding sites (-785/-774) in this region caused an abrupt decline in chicken *PLIN1* promoter activity with the expression of RXRα, which indicates that the -785/-774 site is the crux involved in RXRα-mediated positive activation of the chicken *PLIN1* gene (Figures 4B,C). Subsequently, four biotin-labeled probes were designed for EMSAs to assess the two regions (Figure 5). All four labeled probes could bind to RXRα nucleoprotein to form bands corresponding to a complex, and these bands almost completely disappeared when a molar excess of unlabeled probe was added, indicating that there are multiple RXRα-binding sites in the promoter of the chicken *PLIN1* gene. In addition, the emergence of a supershift band above the complex band after addition of antibody specific to the Myc tag confirmed that the predicted binding sites at -785/-774 are indeed legitimate binding site for chicken RXRα.

The structures and functions of steroids, retinoic acids, vitamin D and thyroid hormone nuclear receptors encoded by a single gene are evolutionarily conserved. As transcription factors, these nuclear receptors can efficiently identify target genes through a conserved DNA-binding domain and regulate the transcription of these genes. RXRα also has a conserved DNA-binding domain and interacts with a hexanucleotide motif (5’-(A/G)G(G/T)TCA) (Belorusova et al., 2016). The RXRα homodimer preferentially binds direct repeats of the hexanucleotide half-site separated by 1 nucleotide, which is called the retinoid X response element (RXRE) (Lee et al., 1993; Castelein et al., 1996; Zhao et al., 2000). In the present study, bioinformatics analysis predicted the following RXRα-binding site in the -785/-774 region of the chicken *PLIN1* promoter: TGCCCTTTGTCCC. Sequence alignment revealed that the putative RXRα-binding site and RXRE sequence are highly similar (10/13 bp) and that the amino acid compositions and domains of mammalian and chicken RXR are relatively conserved (up to 90%, data not shown). In this study, RXRα positively activated the promoter activity of the chicken *PLIN1* gene in a PPARγ-independent manner. Therefore, we predict that RXRα regulates transcription of the chicken *PLIN1* gene by forming a homodimer through binding to the -785/-774 region.

Activation of a specific target gene by RXR homodimers depends on not only the local abundance of RXR, other NRs and related cofactors but also the specificity of the transcriptional response, which is achieved by cooperation between different nuclear receptors or a given NR coupled to other transcription factors (Osz et al., 2019). Therefore, even though multiple RXRα-binding sites were found in -123/-11 of the chicken *PLIN1* gene promoter region, we could not determine the specific regulatory parameters in this region, such as the oligomeric form of RXRα (homodimer or heterodimer) or the presence of specific binding sites.

Our previous studies showed that overexpression of the *PLIN1* gene can enhance lipid accumulation in chicken preadipocytes (Zhou et al., 2012). In the present study, the chicken *PLIN1* gene was directly and positively regulated by RXRα, indicating that RXRα plays a key role in chicken lipid metabolism. Furthermore, RXRα overexpression induced an increase in intracellular lipid accumulation and concomitant upregulation of the expression of adipogenic marker genes in ICP1 preadipocytes (Figure 6). Previous studies have shown that RXR is associated with various biological processes including cell differentiation and death and lipid metabolism (Lefebvre et al., 2010; Gilardi and Desvergne, 2014). For instance, a liver-specific mutation of RXRα altered fatty acid beta-oxidation and hepatocyte lifespan (Wan et al., 2000; Imai et al., 2001), and hepatocyte RXRα deficiency was shown to contribute to alcohol-induced liver damage (Gyamfi et al., 2008). Adipose tissue-specific knockout of RXRα resulted in resistance to diet-induced obesity in mice, owing to impaired adipogenesis and lipolysis (Imai et al., 2001). Interestingly, this function seems to be mediated by RXR homodimers (Nunez et al., 2010). In summary, we demonstrate that RXRα can promote chicken adipogenesis and that this function is at least in part achieved by upregulating *PLIN1* expression.

Taken together, our results revealed (i) a novel RXRα-mediated mechanism by which transcription of the chicken *PLIN1* gene is regulated and (ii) the role of RXRα in adipogenesis, which may allow us to identify novel therapeutic strategies to protect against obesity.

## Data Availability Statement

The datasets generated for this study are available on request to the corresponding author.

## Ethics Statement

The animal study was reviewed and approved by the Institutional Biosafety Committee of the Northeast Agricultural University (Harbin, China).

## Author Contributions

YW conceived and supervised the study. YW, YS, and WZ designed the experiments. YS, GZ, and RL performed the experiments. YL and ZC contributed the reagents and materials. YS analyzed the data and wrote the manuscript. All authors made manuscript revisions.

## Conflict of Interest

The authors declare that the research was conducted in the absence of any commercial or financial relationships that could be construed as a potential conflict of interest.

## Footnotes

1 http://jaspar.genereg.net/

## Supplementary Material

FIGURE S1 Transcriptional activation of the chicken *PLIN1* gene by RXRα is independent of PPARγ. **(A)** Expression levels of the chicken *PPAR*γ gene following transfection with sh-PPARγ. **(B)** Expression levels of the chicken *PLIN1* gene with RXRα overexpression in the interim decreased expression of the chicken *PPAR*γ gene. The pCMV-Myc-RXRα and sh-PPARγ vectors were cotransfected into DF-1 cells. After 48 h of transfection, the mRNA levels of chicken *PLIN1* were determined by real-time RT-PCR and normalized to chicken β*-actin* mRNA levels. **(C)** The effect of RXRα on *PLIN1* promoter activity is independent of PPARγ. DF-1 cells were cotransfected with the chicken *PLIN1* reporter plasmid (pGL3-Plin-1992/-11), pCMV-Myc-RXRα, or the sh-PPARγ vector. After 48 h of transfection, luciferase reporter activity was assayed and is expressed as relative luciferase activity (Fluc/Rluc). All data are expressed as the mean ± SEM (*n* > 3 independent experiments). ^∗∗^*P* < 0.01.
