# Supplementary figures and images for: RXRα Positively Regulates Expression of the Chicken PLIN1 Gene in a PPARγ-Independent Manner and Promotes Adipogenesis
Source: Front Cell Dev Biol. 2020 May 14;8:349. doi: 10.3389/fcell.2020.00349 (PMC7240111; doi:10.3389/fcell.2020.00349)

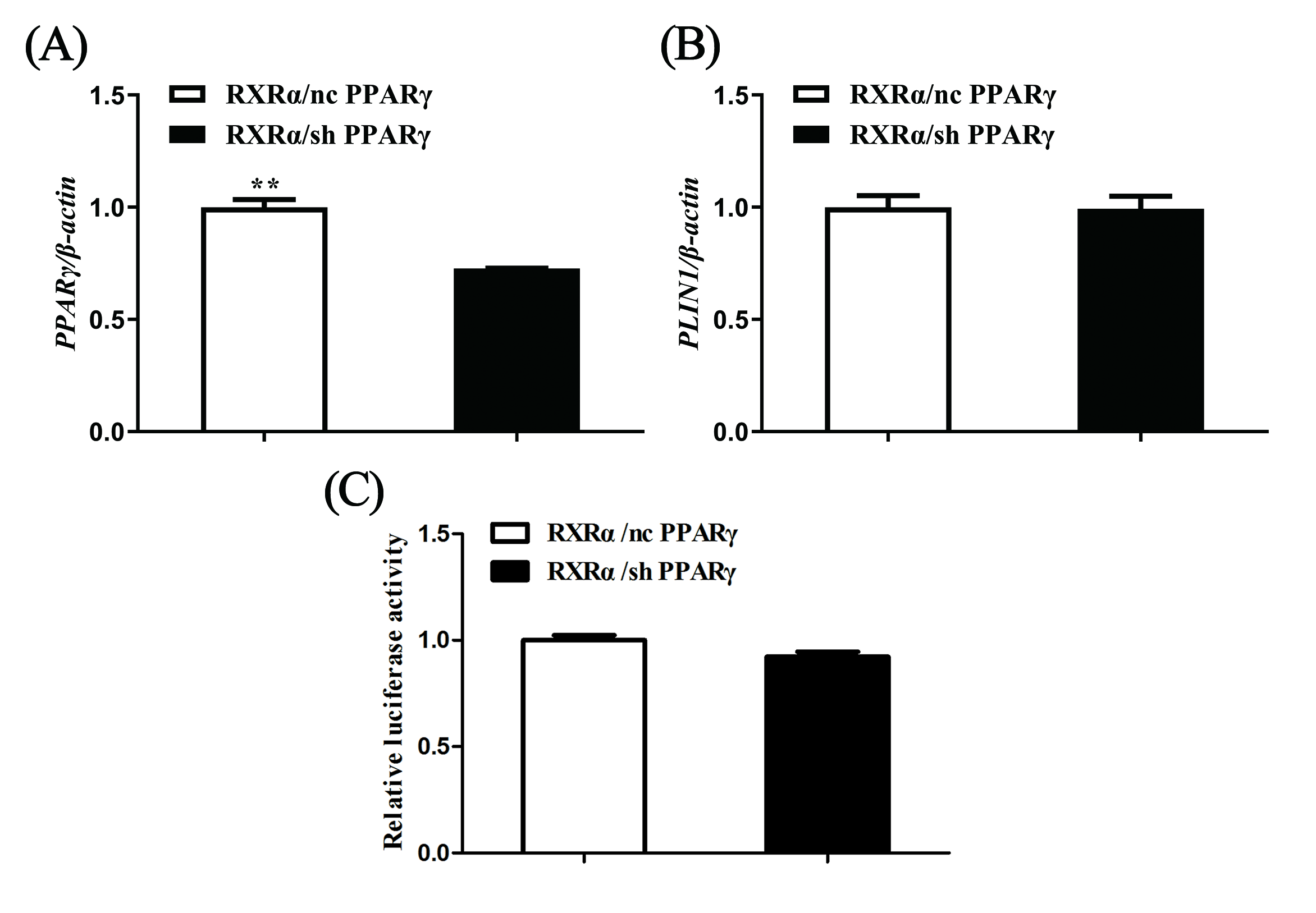

Supplement: FIGURE S1 — Transcriptional activation of the chicken PLIN1 gene by RXRα is independent of PPARγ. (A) Expression levels of the chicken PPARγ gene following transfection with sh-PPARγ. (B) Expression levels of the chicken PLIN1 gene with RXRα overexpression in the interim decreased expression of the chicken PPARγ gene. The pCMV-Myc-RXRα and sh-PPARγ vectors were cotransfected into DF-1 cells. After 48 h of transfection, the mRNA levels of chicken PLIN1 were determined by real-time RT-PCR and normalized to chicken β-actin mRNA levels. (C) The effect of RXRα on PLIN1 promoter activity is independent of PPARγ. DF-1 cells were cotransfected with the chicken PLIN1 reporter plasmid (pGL3-Plin-1992/-11), pCMV-Myc-RXRα, or the sh-PPARγ vector. After 48 h of transfection, luciferase reporter activity was assayed and is expressed as relative luciferase activity (Fluc/Rluc). All data are expressed as the mean ± SEM (n > 3 independent experiments). ∗∗P < 0.01. [file Image_1.TIF]
